# Role of the Extracellular Signal-Regulated Kinase 1/2 Signaling Pathway in Ischemia-Reperfusion Injury

**DOI:** 10.3389/fphys.2019.01038

**Published:** 2019-08-14

**Authors:** Tingting Kong, Minghui Liu, Bingyuan Ji, Bo Bai, Baohua Cheng, Chunmei Wang

**Affiliations:** ^1^Cheeloo College of Medicine, Shandong University, Jinan, China; ^2^School of Mental Health, Neurobiology Key Laboratory of Jining Medical University in Colleges of Shandong, Jining Medical University, Jining, China

**Keywords:** ERK_1/2_ signaling pathway, ischemia-reperfusion injury, protective effect, damage effect, molecular mechanism, therapeutic target

## Abstract

Extracellular signal-regulated kinase 1/2 (ERK_1/2_), an important member of the mitogen-activated protein kinase family, is found in many organisms, and it participates in intracellular signal transduction. Various stimuli induce phosphorylation of ERK_1/2_*in vivo* and *in vitro*. Phosphorylated ERK_1/2_ moves to the nucleus, activates many transcription factors, regulates gene expression, and controls various physiological processes, finally inducing repair processes or cell death. With the aging of the population around the world, the occurrence of ischemia-reperfusion injury (IRI), especially in the brain, heart, kidney, and other important organs, is becoming increasingly serious. Abnormal activation of the ERK_1/2_ signaling pathway is closely related to the development and the metabolic mechanisms of IRI. However, the effects of this signaling pathway and the underlying mechanism differ between various models of IRI. This review summarizes the ERK_1/2_ signaling pathway and the molecular mechanism underlying its role in models of IRI in the brain, heart, liver, kidneys, and other organs. This information will help to deepen the understanding of ERK_1/2_ signals and deepen the exploration of IRI treatment based on the ERK_1/2_ study.

## Introduction

Extracellular signal-regulated kinase 1/2 (ERK_1/2_) is a serine/threonine protein kinase belonging to the mitogen-activated protein kinase (MAPK) family and is widely found in eukaryotic cells. ERK_1/2_ plays a key role in signal transduction from surface receptors to the nucleus. Activated ERK_1/2_ phosphorylates substrates in the cytoplasm or nucleus, and thereby induces expression or activation of specific proteins, leading to regulation of cell proliferation, differentiation, apoptosis, and other processes.

ERK_1/2_ is abnormally expressed in various models of ischemia-reperfusion injury (IRI) (Minutoli et al., 2005; Wang et al., 2006; Zuo et al., 2006). It is the results of the various studies in this area that support an important role of ERK_1/2_ in IRI. ERK_1/2_ has been targeted in an attempt to prevent and treat IRI (Cui et al., 2014). However, the effects of the ERK_1/2_ signaling pathway and the underlying mechanism differ between various models of IRI. These differences are summarized in this review. This information will help to increase understanding of the ERK_1/2_ signaling pathway and to identify a potential therapeutic target for IRI.

## The Extracellular Signal-Regulated Kinase 1/2 Signaling Pathway

### Extracellular Signal-Regulated Kinase 1/2

The *ERK* gene is located on chromosome 1p34~35, has a total length of 3,118 bp, and harbors an open reading frame encoding 978 amino acids. In the early 1990s, Boulton et al. (1990) isolated and identified the cDNA sequence of a protein kinase. This kinase was named ERK_1/2_ because it is activated by various extracellular signals and has a relative molecular weight of 44/42 kDa. The ERK family is divided into five subgroups called ERK_1_–ERK_5_. Among these, ERK_1_ and ERK_2_ are the most widely studied and are generally referred to as ERK_1/2_ because they exhibit 90% homology (Boulton and Cobb, 1991).

### Organization of the Extracellular Signal-Regulated Kinase 1/2 Signaling Pathway

ERK_1/2_ is an important subfamily of the MAPK family and is part of a three-stage enzymatic cascade reaction (Robinson and Cobb, 1997), which comprises Raf as a MAPKKK, MAPK/ERK_1/2_ kinase (MEK) as a MAPKK, and ERK_1/2_ as a MAPK. ERK_1/2_ is normally located in the cytoplasm. Intracellular and extracellular stimuli induce serine/threonine phosphorylation in the VIII region of ERK_1/2_ *via* the classical Ras-Raf-MEK-ERK_1/2_ pathway, which leads to activation of ERK_1/2_. This well-studied pathway is involved in various physiological and pathological processes, such as cell growth, development, proliferation, and differentiation.

Extracellular stimuli, such as growth factors, neurotransmitters, inflammation, ischemia, and hypoxia, stimulate their corresponding receptors and activate tyrosine kinases, and then the signal is transmitted to Ras. Ras-GTP directly binds to Raf and induces its translocation from the cytoplasm to the cell membrane, where it generates a transient membrane-associated signal. Activated Raf activates MEK by phosphorylating serine residues in its catalytic region. MEK activates ERK_1/2_, which phosphorylates cytoplasmic target proteins or regulates the activities of other protein kinases. More importantly, activated ERK_1/2_ enters the nucleus and phosphorylates various transcription factors, such as Elk-l, c-Myc, STATs, Jun, Fos, ATF_2_, and Max. These factors regulate transcription of their target genes and thereby change the expression or activities of specific proteins, ultimately leading to the regulation of cellular metabolism and function (Widmann et al., 1999; Boilly et al., 2000). The classical ERK_1/2_ pathway comprises Ras-Raf-MEK-ERK_1/2_. Many other factors, such as growth factors, cytokines, viruses, G protein-coupled receptor ligands, and oncogenes, also induce phosphorylation of ERK_1/2_. ERK_1/2_ may be activated *via* Gq-PI3K-Raf (Otsuka et al., 2007) and AC-cAMP-PKA-Raf (Otani et al., 2018) signaling. These findings highlight the complexity and importance of ERK_1/2_ signaling pathways.

**Figure 1:**
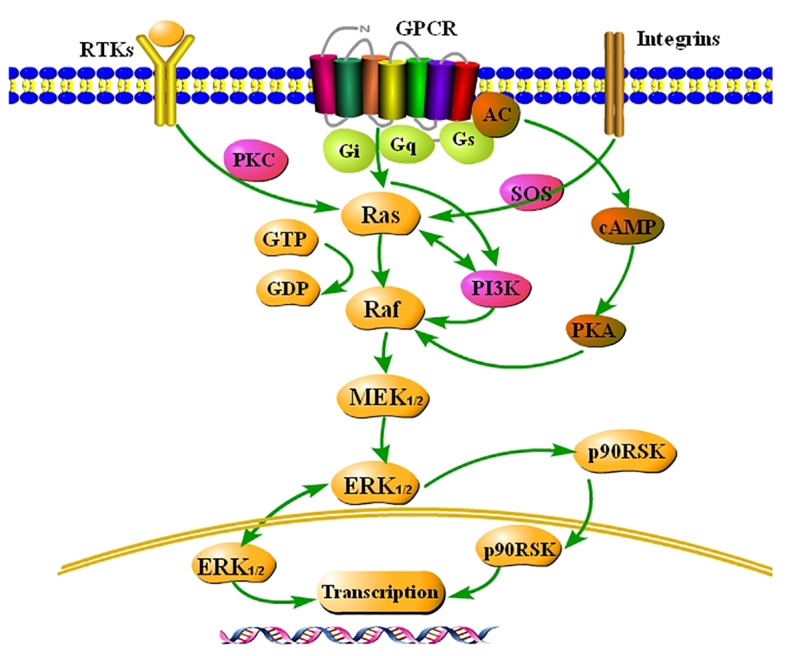
Summary of the classical Ras-Raf-MEK-ERK_1/2_ signaling pathway. Extracellular signals stimulate G-protein-coupled receptors (GCPRs), receptor tyrosine kinases (RTKs), and integrins on the membrane, and activate ERK_1/2_, a key signaling molecule, *via* a series of cascade reactions. ERK_1/2_ is usually located in the cytoplasm. Once activated, ERK_1/2_ rapidly crosses the nuclear membrane, reactivates transcription factors, regulates transcription of target genes, induces expression or activation of specific proteins, and finally regulates cellular metabolism and function.

The Ras-Raf-MEK-ERK_1/2_ signaling pathway is summarized in Figure 1 based on a large amount of literature.

### Functions of the Extracellular Signal-Regulated Kinase 1/2 Signaling Pathway

The functions of the Ras-Raf-MEK-ERK_1/2_ signaling pathway have been extensively studied in recent years. This pathway plays an indispensable role in tumorigenesis and development. Abnormal activation of the Ras-Raf-MEK-ERK_1/2_ signaling pathway can lead to aberrant activation of downstream signaling elements. This affects apoptosis and differentiation of normal cells and promotes their malignant transformation and abnormal proliferation, leading to tumorigenesis (Overmeyer, 2011; Asati et al., 2016; Zaleśna et al., 2016). The Ras-Raf-MEK-ERK_1/2_ signaling pathway also plays an important role in cell cycle regulation. It promotes proliferation by regulating transcription, translation, and degradation of key cell cycle-related proteins (Coleman et al., 2004). Many studies demonstrated that the Ras-Raf-MEK-ERK_1/2_ signaling pathway initiates autophagy by upregulating expression of the autophagy marker proteins LC3 and p62 under stress conditions (Kim et al., 2014). On the other hand, this signaling pathway also inhibits autophagy by downregulating expression of lysosomal-associated membrane proteins 1 and 2, and thereby preventing autophagosome-lysosome fusion (Sivaprasad and Basu, 2008). These results indicate that the ERK_1/2_ signaling pathway performs multiple regulatory functions by activating different targets, which makes it difficult to study the functions of this pathway.

**Figure 2:**
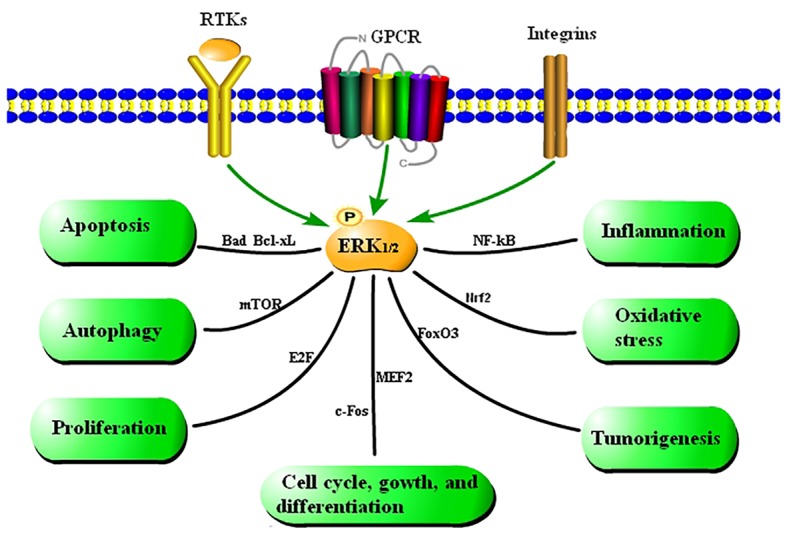
Summary of the functions of the ERK_1/2_ signaling pathway. Cytokines regulate cellular processes *via* the ERK_1/2_ signaling pathway. Extracellular stimulation leads to phosphorylation of ERK_1/2_, which affects various cellular activities by mediating activation of transcription factors.

The functions of the ERK_1/2_ signaling pathway are summarized in Figure 2 based on a large amount of literature.

## Ischemia-Reperfusion Injury

Normal metabolism and functional maintenance of tissues and organs are dependent on normal blood circulation. Local ischemia caused by various factors often leads to injury of tissues and cells. Early restoration of blood flow in ischemic tissue (i.e., reperfusion) is the most effective method to treat ischemic injury. However, reperfusion can aggravate reversible ischemic injury and induce irreversible damage. This effect, called IRI, is involved in the pathogenesis of organ transplantation and various diseases and affects the prognosis of patients with ischemic diseases. Elucidation of the mechanism underlying IRI is important to ameliorate or prevent this condition and to treat ischemic diseases.

The mechanism underlying IRI is very complicated and involves primary injury in the early stage of ischemia and secondary injury after reperfusion. Tissue damage due to IRI not only depends on the degree of blood flow reduction, but is also related to intracellular calcium overload (Hu et al., 2018), oxidative stress, the inflammatory response (Al-Salam and Hashmi, 2018), neurotoxicity of excitatory amino acids (Dohmen et al., 2005), excessive nitric oxide synthesis, energy metabolism disorders, and other factors. Many factors that induce IRI influence each other, leading to neurotoxicity and eventually local brain tissue damage and necrosis/apoptosis of nerve cells.

The relationship between the ERK_1/2_ signaling pathway and IRI in different tissues and organs is discussed in this review.

## The Extracellular Signal-Regulated Kinase 1/2 Signaling Pathway and Ischemia-Reperfusion Injury

### The Extracellular Signal-Regulated Kinase 1/2 Signaling Pathway and Cerebral Ischemia-Reperfusion Injury

Ischemic stroke is a common disease that causes neurological disorders and cognitive impairment (Mori, 2010). Thrombolytic therapy is the most effective treatment for stroke at present; however, it has a strict time window and readily induces Cerebral IRI (CIRI). CIRI is the most common complication of thrombolytic therapy in stroke patients. ERK_1/2_ is widely found in the nervous system. Phosphorylation of ERK_1/2_ is indispensable for its functions, such as inducing proliferation and differentiation, and inhibiting programmed cell death upon brain injury (Roux, 2004). ERK_1/2_ is activated in the early stage after brain injury. The role of the ERK_1/2_ signaling pathway in CIRI has become a hot topic in recent years, however, there are two contradictory views.

**Figure 3:**
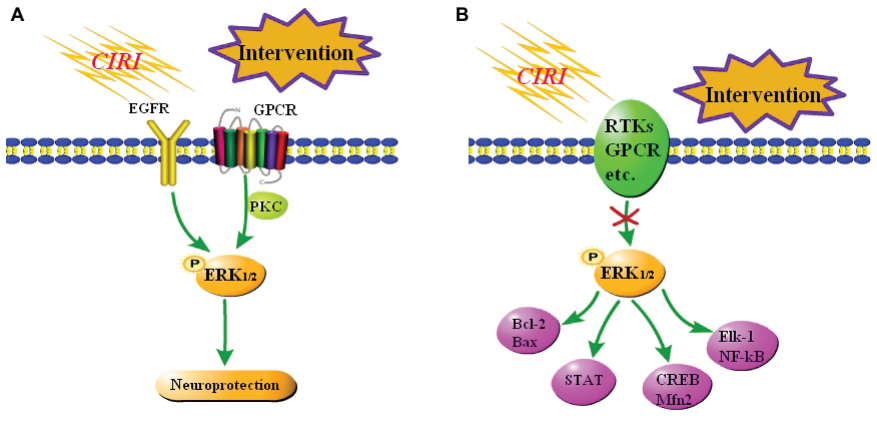
Summary of the role of the ERK_1/2_ signaling pathway in CIRI. **(A)** Neuroprotection against CIRI *via* activation of the ERK_1/2_ signaling pathway. A few studies reported that activation of the ERK_1/2_ signaling pathway elicits neuroprotective effects against CIRI. **(B)** Neuroprotection against CIRI *via* blockade of the ERK_1/2_ signaling pathway. Most studies reported that inhibition of the ERK_1/2_ signaling pathway blocks downstream damage, including inflammation and apoptosis, and thereby protects against CIRI.

Some studies demonstrated that activation of the ERK_1/2_ signaling pathway improves CIRI *in vivo* and *in vitro*. Astragaloside IV effectively activates the EGFR-ERK_1/2_ signaling cascade, and thereby promotes proliferation and neurogenesis of neural stem cells in the brain following transient ischemia and improves nerve function in rats after ischemic stroke (Chen et al., 2018). Protein kinase C (PKC) reduces ischemic injury of mouse cortical neurons by inhibiting ubiquitin C-terminal hydrolase L1 expression, which may negatively regulate autophagy by activating the ERK_1/2_-mTOR signaling pathway (Wang et al., 2017b). Resveratrol protects CA1 neurons from focal CIRI by activating the ERK_1/2_-CREB signaling pathway (Li et al., 2016). α-Phenyl-N-tert-butylnitron induces growth of PC12 neuronal cells by activating the Ras-ERK_1/2_ and PKC signaling pathways (Tsuji and Inanami, 2001). These pathways are summarized in Figure 3A.

However, most reports suggest that activation of the ERK_1/2_ signaling pathway is neurotoxic in focal CIRI models. DL-3-n-butylphthalide elicits a neuroprotective effect against CIRI by inhibiting the ERK_1/2_ signaling pathway and reducing phosphorylation of GRASP65 (Zhu et al., 2018). Combined treatment of CIRI with acupuncture and mild hypothermia may downregulate the phosphorylation levels of MEK-2, ERK_1/2_, and other proteins, increase Bcl-2 expression, and decrease Bax expression, and thereby reduce apoptosis (Lin et al., 2017). Resveratrol inhibits activation of the ERK_1/2_-STAT signaling pathway, effectively reduces the inflammatory response, oxidative stress, and hippocampal neuron loss, and ultimately ameliorates brain injury after CIRI (Chang et al., 2018). At the same time, some studies reported that inhibition of the ERK_1/2_ signaling pathway aggravates CIRI. Nuclear receptor subfamily 4 group A member 1 (NR4A1) inhibits the MAPK-ERK_1/2_-CREB signaling pathway and thereby reduces Mfn2 expression, represses mitophagy, and activates mitochondrial apoptosis in reperfused brains. Upregulation of NR4A1 is a key step in the initiation of CIRI (Zhang and Yu, 2018). The studies *in vitro* have demonstrated that activation of the ERK_1/2_ signaling pathway exacerbates CIRI. Oxygen-glucose deprivation induces vascular endothelial growth factor (VEGF) signaling *via* VEGF receptor 2 (Flk-1) and activates ERK1/2 through an oxidative stress-dependent mechanism. Phosphorylated ERK1/2 activates Elk-1 and other transcription factors, such as NF-κB, and promotes cell death (Narasimhan et al., 2009). These pathways are summarized in Figure 3B.

The pathophysiological mechanisms of CIRI appear to differ between animal models. In addition, the mechanisms underlying hypoxia tolerance in response to different treatment protocols also differ. Therefore, the mechanism underlying the involvement of the ERK_1/2_ signaling pathway in CIRI must be explored further.

### The Extracellular Signal-Regulated Kinase 1/2 Signaling Pathway and Myocardial Ischemia-Reperfusion Injury

Myocardial IRI (MIRI) is a type of myocardial injury caused by restoration of coronary blood flow after ischemia. This eventually leads to death of myocardial cells that initially survived after ischemia and seriously threatens human health (Piper et al., 1998). Frequent heart damage can lead to irreversible fatal injuries and even death (Yndestad et al., 2016). Decades of in-depth research has led to limited understanding of the relationship between the ERK_1/2_ signaling pathway and MIRI. Inflammation is one of the most common features of hypoxia-reoxygenation (H/R) injury in the heart (Yang et al., 2014). Activation of ERK_1/2_ ameliorates acute myocardial infarction in rats by reducing expression of inflammatory cytokines (Duan et al., 2015). Oxidative stress also induces H/R injury of the heart (Vaziri, 2014). The MAPK/ERK_1/2_ signaling pathway has been reported to both aggravate and protect against MIRI (Jiang et al., 2013).

Various drugs elicit therapeutic effects on MIRI by activating the ERK_1/2_ signaling pathway. Carvacrol protects against MIRI by activating various signaling pathways such as MAPK/ERK_1/2_ and thereby reducing apoptosis (Chen et al., 2017). MIRI activates ERK_1/2_ and subsequently the Gsk-3β-p53 and noxa-mcl-1 signaling pathways, which are involved in MIRI. Short-term exposure to TPEN or flavonoids at the beginning of reperfusion may have therapeutic potential. Mammalian STE20-like kinase 1 (Mst1) regulates expression of FUNDC1 through the MAPK/ERK_1/2_-CREB pathway, which reduces mitochondrial apoptosis of cardiomyocytes and improves MIRI (Yu et al., 2019). Prazosin protects cardiomyocytes against inflammation, oxidative stress, and apoptosis by increasing expression and activity of ERK_1/2_ (Wang et al., 2017a). Preconditioning with remifentanil reduces cell injury and death, and protects against H/R injury in adult rat cardiomyocytes. These protective effects are mainly dependent on activation of the PI3K/Akt and ERK_1/2_ signaling pathways. Furthermore, experiments using inhibitors demonstrated that ERK_1/2_ is located downstream of PI3K (Dou et al., 2016).

Other studies suggested that activation of the ERK_1/2_ signaling pathway aggravates MIRI. Activation of ERK_1/2_ has been reported to exacerbate MIRI. Formyl peptide receptor 1 (FPR1) is proinflammatory, contributes to MIRI, and silencing of FPR1 gene have a negative effect on ERK_1/2_ signaling pathway and reduce inflammation, apoptosis, and ventricular remodeling (Zhou et al., 2018). Gypenoside protects cardiomyocytes against MIRI by suppressing activation of NF-κB (p65) *via* inhibition of the ERK_1/2_ signaling pathway *in vitro* and *in vivo*, suggesting that gypenoside can be used to prevent or treat MIRI (Yu et al., 2016). U0126 ameliorates H/R injury by inhibiting the MEK/ERK_1/2_ pathway and downstream EGR-1 expression, which may be a potential approach to alleviate MIRI (Wang et al., 2016).

Further investigation of the relationship between the ERK_1/2_ signaling pathway and MIRI will provide new directions for treatment of this condition.

### The Extracellular Signal-Regulated Kinase 1/2 Signaling Pathway and Hepatic Ischemia-Reperfusion Injury

Hepatic IRI (HIRI) is a common clinical problem observed after liver transplantation, hepatectomy, severe trauma, and hemorrhagic shock, and is caused by sudden restoration of blood supply following hypoxia (Nace et al., 2013; Li et al., 2015). Reperfusion is usually beneficial; however, HIRI is the main cause of organ rejection, dysfunction, and even failure after liver transplantation (Kim et al., 2017), and thus causes significant morbidity and mortality. HIRI involves various factors, including activated Kupffer cells, proinflammatory cytokines, chemokines, adhesion molecules, upregulated oxidation factors, and stress responses (Bilzer and Gerbes, 2000; Van Golen et al., 2013). Apoptosis and necrosis occur during HIRI (Malhi et al., 2006).

The relationship between the ERK_1/2_ signaling pathway and HIRI has been a focus of research in recent years. Various signaling proteins, including members of the MAPK family, are activated upon HIRI (Yang et al., 2008; Li et al., 2015; Feng et al., 2017), while inhibition of the MAPK pathways is generally considered to protect against HIRI. Pretreatment with astaxanthin protects against hepatocyte apoptosis and thus HIRI by inhibiting the ERK_1/2_ signaling pathway and is a therapeutic option (Li et al., 2017). During HIRI, necrosis leads to hepatic damage, which induces autophagy *via* activation of ERK_1/2_. Necrostatin-1 alleviates this damage by reducing expression of ERK_1/2_ (Hong et al., 2016). Propylene glycol alginate sodium sulfate protects against HIRI by inhibiting several pathways, including the ERK_1/2_ signaling pathway, and reducing inflammation, apoptosis, and autophagy (Xu et al., 2017). Pretreatment with salidroside is a therapeutic option that protects hepatocytes against HIRI in mice by inhibiting the ERK_1/2_ signaling pathway and thereby decreasing inflammation, apoptosis, and autophagy in the liver (Feng et al., 2017).

According to these studies, various drugs protect against HIRI by modulating the ERK_1/2_ signaling pathway. Further investigation of the underlying mechanism may help to develop a new therapy for HIRI.

### The Extracellular Signal-Regulated Kinase 1/2 Signaling Pathway and Renal Ischemia-Reperfusion Injury

Renal IRI (RIRI) is characterized by restriction of renal blood flow and subsequent restoration of normal blood flow and oxygen supply. This aggravates renal damage by increasing inflammation *via* modulation of various cytokines and reactive oxygen species (ROS) (Wahhabaghai et al., 2015), and by modulating cellular function (Khader et al., 2015). Acute ischemia damages renal cells. After reperfusion, the initial injury is aggravated by a cascade of inflammatory reactions, such as inflammatory cell activation, cytokine secretion, and other inflammatory reactions, which induce apoptosis or necrosis of renal parenchymal cells (Bonventre and Zuk, 2004). RIRI causes renal insufficiency and high mortality (Ma et al., 2009), and has adverse effects on ~7–20% of hospitalized patients (Wang et al., 2012). This condition is caused by a series of complicated events (Hashemi, 2014). ERK_1/2_ is required to repair renal tubular epithelial cells and inhibit fibrosis caused by renal injury (Jang et al., 2013), suggesting that the ERK_1/2_ signaling pathway is related to the pathophysiology of various renal diseases, including RIRI (Kumar et al., 2009).

Research on RIRI in which Clusterin (CLU) participates revealed that a complex signaling network, which includes the PI3K/Akt, VEGF, and MAPK/ERK1/2 signaling pathways, mediates survival and proliferation of renal cells (Dairi et al., 2016). Inhibition of the ERK_1/2_ signaling pathway was reported to elicit beneficial effects on RIRI in most studies. Dexamethasone induces acetylation of the p65 subunit of NF-κB by inhibiting MAPK/ERK_1/2_ activation and subsequent translocation of HMGB1, leading to attenuation of inflammation in RIRI (Zhang et al., 2016). Stanniocalcin-1 protects against renal injury and reduces inflammation, oxidation, and apoptosis by inhibiting ROS-mediated expression of various genes encoding components of the PKC-ERK_1/2_-NF-κB pathway (Liu et al., 2016a). C-reactive protein interacts with Fcγ receptor II and activates Smad3 *via* ERK_1/2_/p38 to promote acute kidney injury. Gene deletion or drug inhibition of Smad3 blocks this pathway, and thereby improves acute kidney injury (Lai et al., 2016). Rapid and sustained perturbation of mitochondrial homeostasis is important in RIRI (Zhang and Yu, 2018). Pretreatment with trametinib blocks ERK_1/2_ phosphorylation upon RIRI and attenuates downregulation of PGC-1α and its downstream target genes, thereby reducing mitochondrial biosynthesis (Collier et al., 2016). In contrast, activation of the ERK_1/2_ pathway has been suggested to elicit beneficial effects on RIRI. Inhibition of ERK_1/2_ phosphorylation increases apoptosis and oxidative stress in hypothermic kidneys following ischemia-reperfusion. Hypothermia induces ERK_1/2_ phosphorylation and thereby reduces renal damage, renal cell apoptosis, and oxidative stress induced by RIRI (Choi et al., 2017).

Further investigation of these pathways is required and may help to develop a new strategy to treat or prevent RIRI.

### The Extracellular Signal-Regulated Kinase 1/2 Signaling Pathway and Lung Ischemia-Reperfusion Injury

Lung transplantation provides hope for many patients with end-stage lung disease. Acute lung injury (ALI) induced by lung IRI (LIRI) is an important problem following lung transplantation (Xu et al., 2011). During reperfusion, blood and oxygen are reintroduced into the ischemic pulmonary parenchyma and create a toxic environment by inducing production of ROS, activation of the immune and coagulation systems, endothelial dysfunction, and death of apoptotic cells (Weyker et al., 2013). Consequently, pulmonary function is seriously perturbed. ALI induced by LIRI is the main reason for lung transplantation failure and is an important cause of early death after lung transplantation (Tsuang et al., 2013). ERK_1/2_ is activated during human lung transplantation (Sakiyama et al., 2005) and promotes apoptosis (Wada and Penninger, 2004; Lu and Xu, 2006).

Most studies reported that activation of the ERK_1/2_ signaling pathway is involved in development of LIRI; therefore, inhibition of this pathway may alleviate LIRI. Hypoxia and inflammation induce VEGF production (Wu et al., 2013), and VEGF increases the severity of lung injury induced by LIRI. Expression of VEGF and downstream ERK_1/2_ are increased in patients with ALI induced by LIRI. Pretreatment with an anti-VEGF antibody significantly inhibits VEGF and ERK_1/2_ expression, and alleviates lung injury induced by LIRI (Lan et al., 2016). Activation of the ERK_1/2_ signaling pathway induces severe LIRI, which leads to inflammation, edema, and neutrophil infiltration, while inhibition of ERK_1/2_ significantly reduces LIRI and thus lung injury (Masanori et al., 2018).

Based on these findings, inhibition of the ERK_1/2_ signaling pathway is a potential therapeutic strategy for LIRI.

### The Extracellular Signal-Regulated Kinase 1/2 Signaling Pathway and Intestinal Ischemia-Reperfusion Injury

Intestinal IRI (IIRI) is a well-known risk factor for clinical morbidity and causes delayed dysfunction due to induction of ischemia and subsequent activation of the inflammatory pathway (Mallick et al., 2007; Kocogullari et al., 2008). Severe intestinal ischemia can result in loss of intestinal epithelium, increased intestinal permeability, sepsis, and multiple organ failure (Cai et al., 2012). Despite recent improvements in diagnosis and interventional therapy, mortality of patients with IIRI remains surprisingly high (Brandt and Boley, 2000). Therefore, the mechanism underlying IIRI must be clarified to improve treatment. Early recovery of intestinal function after IIRI occurs within 3 h of reperfusion and is a complicated process (Elassal and Besner, 2005). Restoration of the original state is key to reestablish intestinal barrier function in the early phase after trauma. This begins as early as 15 min after injury and is consistent with continued tissue damage during reperfusion, in contrast with other types of healing (Takahashi et al., 1995; Wilson and Gibson, 1997). Therefore, a portion of the intestinal mucosa must survive upon IIRI and undergo repair to restore the original state.

Various studies reported that activation of the ERK_1/2_ signaling pathway plays an important role in improvement of IIRI and is beneficial for repair. IIRI results in tissue damage, loss of visceral barrier function, and distant complications, including multiple organ dysfunction syndrome. Reperfusion induces activation of PI3K/Akt and MEK/ERK_1/2_, which are involved in recovery, leading to restoration of tissue and barrier function. Endogenous HB-EGF is induced after IIRI, which may activate PI3K/Akt and MEK/ERK_1/2_ *via* ErbB1 to improve IIRI and facilitate recovery of intestinal barrier function (Elassal and Besner, 2005). Another study reported that keratinocyte growth factor treatment reduces apoptosis of intestinal epithelial cells exposed to hypoxia by activating the Akt/ERK_1/2_ signaling pathway and increasing E-cadherin expression (Cai et al., 2018).

However, there are opposing studies suggesting that ERK_1/2_ contributes to IIRI, Chen et al. showed that exogenous BMP2/4 can activate the ERK_1/2,_ which contributes to NF-kB activation and increases inflammation during IIRI (Chen et al., 2014).

These findings increase understanding of IIRI and may help to develop a new therapeutic approach for this condition.

### The Extracellular Signal-Regulated Kinase 1/2 Signaling Pathway and Spinal Cord Ischemia-Reperfusion Injury

Spinal cord IRI (SIRI) occurs during surgical repair of thoracic and celiac aneurysms resulting from temporary aortic occlusion. SIRI can lead to spinal cord dysfunction and paraplegia, which is the most serious complication of thoracic and abdominal aortic surgery, with a reported incidence of higher than 10% (Greenberg et al., 2008). SIRI has long been recognized as a clinical problem. At present, nutritional support, enhancement of blood resistance, and early postoperative monitoring are mainly used to improve spinal cord function. Unfortunately, these strategies are largely ineffective in paraplegic patients and impose considerable economic burdens on the individual patient and society as a whole. Therefore, new approaches are urgently needed to prevent and treat SIRI.

Most studies reported that activation of the ERK_1/2_ signaling pathway protects against SIRI. Both standard and modified distal posterior adaptation elicits neuroprotective effects against spinal cord ischemia by increasing expression of p-Akt and p-ERK_1/2_ (Jiang et al., 2009). Xenon-delayed post-processing improves neurological function and attenuates SIRI in rats by increasing p-Akt and p-ERK_1/2_ expression, and activating the Akt and ERK_1/2_ signaling pathways (Liu et al., 2016b).

SIRI causes great suffering to patients and imposes a great burden on their families and society. Therefore, the mechanism underlying SIRI must be urgently studied, and methods to prevent and treat this condition must be developed. Further investigation of the ERK_1/2_ signaling pathway may help to identify a new therapeutic approach for SIRI.

## Future Prospects

The ERK_1/2_ pathway is one of the most important signal transduction pathways and regulates many biological responses. It even plays a key role in behavioral responses and cognitive processes, such as learning and memory. ERK_1/2_ is abnormally expressed upon IRI *in vivo* and *in vitro*. Even transient local ischemia can change ERK_1/2_ expression and induce corresponding effects.

The period, degree, and location of injury may determine whether the ERK_1/2_ signaling pathway protects against or aggravates IRI. This pathway elicits different effects, including inhibition of apoptosis and induction of necrosis, depending on the period of ischemic injury and tissue region. The mechanism underlying these effects must be investigated further.

Abnormalities in the ERK_1/2_ signaling pathway can induce many pathological changes, such as tumorigenesis. Consequently, many MEK and ERK_1/2_ inhibitors, such as the non-steroidal anti-inflammatory drug NS398, have been developed. This drug blocks the Ras/c-Raf interaction and inhibits activation of the ERK_1/2_ signaling pathway (Pan et al., 2008), thereby inactivating matrix metalloproteinase-2 and inhibiting growth and metastasis of cancer cells. Unfortunately, such inhibitors have many side effects due to the regulatory complexity of the ERK_1/2_ signaling pathway. Investigation of the regulatory mechanism of this pathway will help to identify proteins that directly regulate ERK_1/2_ and to determine their involvement in ERK_1/2_-related diseases. This approach will facilitate the development of drugs with improved targeting and fewer side effects.

This review summarizes the role of the ERK_1/2_ signaling pathway in IRI and the underlying mechanisms. The effect of the ERK_1/2_ signaling pathway on IRI remains controversial. Further comprehensive and in-depth studies are required to determine whether the ERK_1/2_ signaling pathway protects against or aggravates IRI. Elucidation of the underlying mechanisms will help to establish a theoretical basis for the clinical treatment of IRI.

## Author Contributions

TK wrote the manuscript and generated the figures. ML helped to design the figures. BJ and BB proposed suggestions for revisions. BC revised the manuscript. CW designed the content and provided intellectual insight.

### Conflict of Interest Statement

The authors declare that the research was conducted in the absence of any commercial or financial relationships that could be construed as a potential conflict of interest.

## References

[ref1] Al-Salam S. Hashmi S. (2018). Myocardial ischemia reperfusion injury: apoptotic, inflammatory and oxidative stress role of galectin-3. Cell. Physiol. Biochem. 50, 1123–1139. 10.1159/000494539, PMID: 30355930

[ref2] Asati V. Mahapatra D. K. Bharti S. K. (2016). Pi3k/akt/mtor and ras/raf/mek/erk signaling pathways inhibitors as anticancer agents: structural and pharmacological perspectives. Eur. J. Med. Chem. 109, 314–341. 10.1016/j.ejmech.2016.01.012, PMID: 26807863

[ref3] Bilzer M. Gerbes A. L. (2000). Preservation injury of the liver: mechanisms and novel therapeutic strategies. J. Hepatol. 32, 508–515. 10.1016/s0168-8278(00)80404-3 10735623

[ref4] Boilly B. Vercoutteredouart A. Hondermarck H. Nurcombe V. Bourhis X. L. (2000). FGF signals for cell proliferation and migration through different pathways. Cytokine Growth Factor Rev. 11, 295–302. 10.1016/S1359-6101(00)00014-9, PMID: 10959077

[ref5] Bonventre J. V. Zuk A. (2004). Ischemic acute renal failure: an inflammatory disease? Kidney Int. 66, 480–485. 10.1111/j.1523-1755.2004.761_2.x, PMID: 15253693

[ref6] Boulton T. G. Cobb M. H. (1991). Identification of multiple extracellular signal-regulated kinases (erks) with antipeptide antibodies. Cell Regul. 2, 357–371. 10.1091/mbc.2.5.357, PMID: 1654126 PMC361802

[ref7] Boulton T. Yancopoulos G. Gregory J. Slaughter C. Moomaw C. Hsu J.. (1990). An insulin-stimulated protein kinase similar to yeast kinases involved in cell cycle control. Science 249, 64–67. 10.1126/science.2164259, PMID: 2164259

[ref8] Brandt L. J. Boley S. J. (2000). Aga technical review on intestinal ischemia. American gastrointestinal association. Gastroenterology 118, 954–968. 10.1016/S0016-5085(00)70183-1 10784596

[ref9] Cai Y. Wang W. Liang H. Sun L. Teitelbaum D. H. Yang H. (2012). Keratinocyte growth factor improves epithelial structure and function in a mouse model of intestinal ischemia/reperfusion. PLoS One 7:e44772. 10.1371/journal.pone.0044772, PMID: 23028616 PMC3441439

[ref10] Cai Y. Wang W. Qiu Y. Yu M. Yin J. Yang H.. (2018). KGF inhibits hypoxia-induced intestinal epithelial cell apoptosis by upregulating akt/erk pathway-dependent e-cadherin expression. Biomed. Pharmacother. 105, 1318–1324. 10.1016/j.biopha.2018.06.091, PMID: 30021369

[ref11] Chang C. Zhao Y. Song G. She K. (2018). Resveratrol protects hippocampal neurons against cerebral ischemia-reperfusion injury via modulating jak/erk/stat signaling pathway in rats. J. Neuroimmunol. 315, 9–14. 10.1016/j.jneuroim.2017.11.015 29306408

[ref12] Chen Y. Ba L. Huang W. Liu Y. Pan H. Mingyao E.. (2017). Role of carvacrol in cardioprotection against myocardial ischemia/reperfusion injury in rats through activation of MAPK/ERK and Akt/eNOS signaling pathways. Eur. J. Pharmacol. 796, 90–100. 10.1016/j.ejphar.2016.11.053, PMID: 27916558

[ref13] Chen X. Wu H. Chen H. S. Wang Q. Xie X. J. Shen J. G. (2018). Astragaloside VI promotes neural stem cell proliferation and enhances neurological function recovery in transient cerebral ischemic injury via activating EGFR/MAPK signaling cascades. Mol. Neurobiol. 56, 3053–3067. 10.1007/s12035-018-1294-3 30088176

[ref14] Chen K. Xie W. Luo B. Xiao W. Teitelbaum D. H. Zhang C. (2014). Intestinal mucosal barrier is injured by BMP2/4 via activation of NF-κB signals after ischemic reperfusion. Mediat. Inflamm. 2014:901530. 10.1155/2014/901530, PMID: 25132736 PMC4124715

[ref15] Choi D. E. Jeong J. Y. Choi H. Chang Y. K. Ahn M. S. Ham Y. R.. (2017). Erk phosphorylation plays an important role in the protection afforded by hypothermia against renal ischemia-reperfusion injury. Surgery 161, 444–452. 10.1016/j.surg.2016.07.028, PMID: 27590616

[ref16] Coleman M. L. Marshall C. J. Olson M. F. (2004). Ras and rho gtpases in g1-phase cell-cycle regulation. Nat. Rev. Mol. Cell Biol. 5, 355–366. 10.1038/nrm1365 15122349

[ref17] Collier J. B. Whitaker R. M. Eblen S. T. Schnellmann R. G. (2016). Rapid renal regulation of peroxisome proliferator-activated receptor γ coactivator-1α by extracellular signal-regulated kinase 1/2 in physiological and pathological conditions. J. Biol. Chem. 291, 26850–26859. 10.1074/jbc.M116.754762, PMID: 27875304 PMC5207191

[ref18] Cui H. Li X. Li N. Qi K. Li Q. Jin C.. (2014). Induction of autophagy by tongxinluo through the mek/erk pathway protects human cardiac microvascular endothelial cells from hypoxia/reoxygenation injury. J. Cardiovasc. Pharmacol. 64, 180–190. 10.1097/FJC.0000000000000104, PMID: 24705173

[ref19] Dairi G. Guan Q. Roshan-Moniri M. Collins C. C. Ong C. J. Gleave M. E.. (2016). Transcriptome-based analysis of molecular pathways for clusterin functions in kidney cells. J. Cell. Physiol. 231, 2628–2638. 10.1002/jcp.25415, PMID: 27155085

[ref20] Dohmen C. Kumura E. Rosner G. Heiss W. D. Graf R. (2005). Extracellular correlates of glutamate toxicity in short-term cerebral ischemia and reperfusion: a direct in vivo comparison between white and gray matter. Brain Res. 1037, 43–51. 10.1016/j.brainres.2004.12.046, PMID: 15777751

[ref21] Dou M. Wu H. Zhu H. Jin S. Zhang Y. He S. (2016). Remifentanil preconditioning protects rat cardiomyocytes against hypoxia-reoxygenation injury via δ-opioid receptor mediated activation of PI3K/Akt and ERK pathways. Euro. J. Pharmacol. 789, 395–401. 10.1016/j.ejphar.2016.08.002, PMID: 27492364

[ref22] Duan J. Yang Y. Liu H. Dou P. C. Tan S. Y. (2015). Osthole ameliorates acute myocardial infarction in rats by decreasing the expression of inflammatory- related cytokines, diminishing MMP-2 expression and activating p-ERK. Int. J. Mol. Med. 37, 207–216. 10.3892/ijmm.2015.2402 26549213

[ref23] Elassal O. Besner G. (2005). HB-EGF enhances restitution after intestinal ischemia/reperfusion via pi3k/akt and mek/erk1/2 activation. Gastroenterology 129, 609–625. 10.1016/j.gastro.2005.05.054, PMID: 16083716

[ref24] Feng J. Zhang Q. Mo W. Wu L. Li S. Li J. (2017). Salidroside pretreatment attenuates apoptosis and autophagy during hepatic ischemia- reperfusion injury by inhibiting the mitogen-activated protein kinase pathway in mice. Drug Des. Devel. Ther. 11, 1989–2006. 10.2147/DDDT.S136792 PMC5501634 28721018

[ref25] Greenberg R. K. Lu Q. Roselli E. E. Svensson L. G. Moon M. C. Hernandez A. V. (2008). Contemporary analysis of descending thoracic and thoracoabdominal aneurysm repair: a comparison of endovascular and open techniques. Circulation 118, 808–817. 10.1161/CIRCULATIONAHA.108.769695 18678769

[ref26] Hashemi M. (2014). The study of pentoxifylline drug effects on renal apoptosis and bcl-2 gene expression changes following ischemic reperfusion injury in rat. Iran. J. Pharm. Res. 13, 181–189. 10.2329/perio.48.255, PMID: 24734070 PMC3985231

[ref27] Hong J. M. Kim S. J. Lee S. M. (2016). Role of necroptosis in autophagy signaling during hepatic ischemia and reperfusion. Toxicol. Appl. Pharmacol. 308, 1–10. 10.1016/j.taap.2016.08.010, PMID: 27521978

[ref28] Hu S. Zhu P. Zhou H. Zhang Y. Chen Y. (2018). Melatonin-induced protective effects on cardiomyocytes against reperfusion injury partly through modulation of IP3R and SERCA2a via activation of ERK1. Arq. Bras. Cardiol. 110, 44–51. 10.5935/abc.20180008, PMID: 29538523 PMC5831301

[ref29] Jang H. S. Han S. J. Kim J. I. Lee S. Lipschutz J. H. Park K. M. (2013). Activation of erk accelerates repair of renal tubular epithelial cells, whereas it inhibits progression of fibrosis following ischemia/reperfusion injury. Biochim. Biophys. Acta 1832, 1998–2008. 10.1016/j.bbadis.2013.07.001 23851027

[ref30] Jiang X. Ai C. Shi E. Nakajima Y. Ma H. (2009). Neuroprotection against spinal cord ischemia-reperfusion injury induced by different ischemic postconditioning methods: roles of phosphatidylinositol 3-kinase-akt and extracellular signal-regulated kinase. Anesthesiology 111, 1197–1205. 10.1097/ALN.0b013e3181bf1d93, PMID: 19934862

[ref31] Jiang M. Wang L. Jiang H. H. (2013). Role of spinal MAPK-ERK signal pathway in myocardial ischemia-reperfusion injury. Zhongguo Dang Dai Er Ke Za Zhi 15, 387–391. PMID: . 10.7499/j.issn.1008-8830.2013.05.017 23676945

[ref32] Khader A. Yang W. Kuncewitch M. Prince J. M. Marambaud P. Nicastro J.. (2015). Novel resveratrol analogues attenuate renal ischemic injury in rats. J. Surg. Res. 193, 807–815. 10.1016/j.jss.2014.08.015, PMID: 25214260 PMC4268227

[ref33] Kim J. H. Hong S. K. Wu P. K. Richards A. L. Jackson W. T. Park J. I. (2014). Raf/mek/erk can regulate cellular levels of lc3b and sqstm1/p62 at expression levels. Exp. Cell Res. 327, 340–352. 10.1016/j.yexcr.2014.08.001, PMID: 25128814 PMC4164593

[ref34] Kim J. Y. Lee D. Y. Kang S. Miao W. Kim H. Lee Y.. (2017). Bilirubin nanoparticle preconditioning protects against hepatic ischemia-reperfusion injury. Biomaterials 133, 1–10. 10.1016/j.biomaterials.2017.04.011, PMID: 28414974

[ref35] Kocogullari C. U. Becit N. Erkut B. Keles M. S. Ceviz M. Ates A.. (2008). Prevention of reperfusion injury of the spinal cord in aortic surgery: an experimental study. Surg. Today 38, 237–244. 10.1007/s00595-007-3614-5, PMID: 18306998

[ref36] Kumar S. Allen D. A. Kieswich J. E. Patel N. S. A. Harwood S. Mazzon E. (2009). Dexamethasone ameliorates renal ischemia-reperfusion injury. J. Am. Soc. Nephrol. 20, 2412–2425. 10.1681/asn.2008080868 19797168 PMC2799171

[ref37] Lai W. Tang Y. Huang X. R. Ming-Kuen Tang P. Xu A. Szalai A. J.. (2016). C-reactive protein promotes acute kidney injury via Smad3-dependent inhibition of CDK2/cyclin E. Kidney Int. 90, 610–626. 10.1016/j.kint.2016.06.010, PMID: 27470679 PMC5672807

[ref38] Lan C. Peng C. Tang S. Wu S. Huang K. Wu C. (2016). Anti-vascular endothelial growth factor antibody suppresses ERK and NF-κB activation in ischemia-reperfusion lung injury. PLoS One 11:e0159922. 10.1371/journal.pone.0159922, PMID: 27513332 PMC4981443

[ref39] Li Z. Fang F. Wang Y. Wang L. (2016). Resveratrol protects ca1 neurons against focal cerebral ischemic reperfusion-induced damage via the erk-creb signaling pathway in rats. Pharmacol. Biochem. Behav. 146, 21–27. 10.1016/j.pbb.2016.04.007 27143440

[ref40] Li S. Takahara T. Fujino M. Fukuhara Y. Takahara S. (2017). Astaxanthin prevents ischemia-reperfusion injury of the steatotic liver in mice. PLoS One 12:e0187810. 10.1371/journal.pone.0187810, PMID: 29121675 PMC5679630

[ref41] Li J. Wang F. Xia Y. Dai W. Chen K. Li S.. (2015). Astaxanthin pretreatment attenuates hepatic ischemia reperfusion-induced apoptosis and autophagy via the ros/mapk pathway in mice. Mar. Drugs. 13, 3368–3387. 10.3390/md13063368, PMID: 26023842 PMC4483634

[ref42] Lin Y. Liu Q. Chen C. Chen W. Xiao H. Yang Q.. (2017). Effect of acupuncture combined with hypothermia on mapk/erk pathway and apoptosis related factors in rats with cerebral ischemia reperfusion injury. J. Cent. South Univ. 42, 380–388. 10.11817/j.issn.1672-7347.2017.04.003, PMID: 28490694

[ref43] Liu D. Shang H. Liu Y. (2016a). Stanniocalcin-1 protects a mouse model from renal ischemia-reperfusion injury by affecting ros-mediated multiple signaling pathways. Int. J. Mol. Sci. 17:1051. 10.3390/ijms17071051 PMC4964427 27420048

[ref44] Liu S. Yang Y. Jin M. Hou S. Cheng W. (2016b). Xenon-delayed postconditioning attenuates spinal cord ischemia/reperfusion injury through activation akt and erk signaling pathways in rats. J. Neurol. Sci. 368, 277–284. 10.1016/j.jns.2016.07.009 27538649

[ref45] Lu Z. Xu S. (2006). Erk1/2 map kinases in cell survival and apoptosis. IUBMB Life 58, 621–631. 10.1080/15216540600957438 17085381

[ref46] Ma D. Lim T. Xu J. Tang H. Wan Y. Zhao H.. (2009). Xenon preconditioning protects against renal ischemic-reperfusion injury via hif-1α activation. J. Am. Soc. Nephrol. 20, 713–720. 10.1681/ASN.2008070712, PMID: 19144758 PMC2663824

[ref47] Malhi H. Gores G. J. Lemasters J. J. (2006). Apoptosis and necrosis in the liver: a tale of two deaths? Hepatology 43, S31–S44. 10.1002/hep.21062, PMID: 16447272

[ref48] Mallick I. H. Seifalian A. M. Yang W. Winslet M. C. (2007). Ischemia- reperfusion injury of the intestine and protective strategies against injury. Dig. Dis. Sci. 49, 1359–1377. 10.1093/rpd/ncm395 15481305

[ref49] Masanori O. Masaomi Y. Sumiharu Y. Shinji O. Kentaroh M. Seiichiro S.. (2018). Spred2 deficiency may lead to lung ischemia–reperfusion injury via erk1/2 signaling pathway activation. Surg. Today 48, 1089–1095. 10.1007/s00595-018-1696-x, PMID: 30022248

[ref50] Minutoli L. Antonuccio P. Romeo C. Nicotina P. A. Bitto A. Arena S.. (2005). Evidence for a role of mitogen-activated protein kinase 3/mitogen- activated protein kinase in the development of testicular ischemia-reperfusion injury. Biol. Reprod. 73, 730–736. 10.1095/biolreprod.105.040741, PMID: 15944243

[ref51] Mori E. (2010). Impact of subcortical ischemic lesions on behavior and cognition. Ann. N. Y. Acad. Sci. 977, 141–148. 10.1111/j.1749-6632.2002.tb04809.x 12480744

[ref52] Nace G. W. Huang H. Klune J. R. Eid R. E. Rosborough B. R. Korff S.. (2013). Cellular-specific role of toll-like receptor 4 in hepatic ischemia- reperfusion injury in mice. Hepatology 58, 374–387. 10.1002/hep.26346, PMID: 23460269 PMC3688695

[ref53] Narasimhan P. Liu J. Song Y. S. Massengale J. L. Chan P. H. (2009). VEGF stimulates the ERK1/2 signaling pathway and apoptosis in cerebral endothelial cells after ischemic conditions. Stroke 40, 1467–1473. 10.1161/STROKEAHA.108.534644, PMID: 19228841 PMC2663599

[ref54] Otani T. Matsuda M. Mizokami A. Kitagawa N. Takeuchi H. Jimi E.. (2018). Osteocalcin triggers Fas/FasL-mediated necroptosis in adipocytes via activation of p300. Cell Death Dis. 9:1194. 10.1038/s41419-018-1257-7, PMID: 30546087 PMC6294257

[ref55] Otsuka M. Negishi Y. Aramaki Y. (2007). Involvement of phosphatidylinositol-3-kinase and erk pathways in the production of tgf-β1 by macrophages treated with liposomes composed of phosphatidylserine. FEBS Lett. 581, 325–330. 10.1016/j.febslet.2006.12.032, PMID: 17222412

[ref56] Overmeyer J. H. (2011). Death pathways triggered by activated ras in cancer cells. Front. Biosci. 16, 1693–1713. 10.2741/3814, PMID: 21196257 PMC3098755

[ref57] Pan M. Chang H. Hung W. (2008). Non-steroidal anti-inflammatory drugs suppress the ERK signaling pathway via block of Ras/c-Raf interaction and activation of MAP kinase phosphatases. Cell. Signal. 20, 1134–1141. 10.1016/j.cellsig.2008.02.004, PMID: 18374541

[ref58] Piper H. M. Garcnadorado D. Ovize M. (1998). A fresh look at reperfusion injury. Cardiovasc. Res. 38, 291–300. 10.1016/s0008-6363(98)00033-9, 9709390

[ref59] Robinson M. Cobb M. H. (1997). Mitogen-activated protein kinase pathways. Curr. Opin. Cell Biol. 9, 180–186. 10.1016/s0955-0674(97)80061-0 9069255

[ref60] Roux P. P. (2004). ERK and p38 MAPK-activated protein kinases: a family of protein kinases with diverse biological functions. Microbiol. Mol. Biol. Rev. 68, 320–344. 10.1128/MMBR.68.2.320-344.2004, PMID: 15187187 PMC419926

[ref61] Sakiyama S. Hamilton J. Han B. Jiao Y. Shen-Tu G. Perrot M. D.. (2005). Activation of mitogen-activated protein kinases during human lung transplantation. J. Heart Lung Transplant. 24, 2079–2085. 10.1016/j.healun.2005.04.011, PMID: 16364853

[ref62] Sivaprasad U. Basu A. (2008). Inhibition of erk attenuates autophagy and potentiates tumour necrosis factor-α-induced cell death in mcf-7 cells. J. Cell. Mol. Med. 12, 1265–1271. 10.1111/j.1582-4934.2008.00282.x, PMID: 18266953 PMC3865671

[ref63] Takahashi M. Ota S. Shimada T. Hamada E. Kawabe T. Okudaira T.. (1995). Hepatocyte growth factor is the most potent endogenous stimulant of rabbit gastric epithelial cell proliferation and migration in primary culture. J. Clin. Invest. 95, 1994–2003. 10.1172/JCI117884, PMID: 7738166 PMC295775

[ref64] Tsuang W. M. Vock D. M. Ashley F. C. C. Lederer D. J. Palmer S. M. (2013). An acute change in lung allocation score and survival after lung transplantation. Ann. Intern. Med. 158, 650–657. 10.7326/0003-4819-158-9-201305070-00004, PMID: 23648947 PMC3819715

[ref65] Tsuji M. Inanami O. (2001). Induction of neurite outgrowth in PC12 cells by alpha -phenyl-N-tert-butylnitron through activation of protein kinase C and the Ras- extracellular signal-regulated kinase pathway. J. Biol. Chem. 276, 32779–32785. 10.1074/jbc.M101403200, PMID: 11438521

[ref66] Van Golen R. F. Reiniers M. J. Olthof P. B. Van Gulik T. M. Heger M. (2013). Sterile inflammation in hepatic ischemia/reperfusion injury: present concepts and potential therapeutics. J. Gastroenterol. Hepatol. 28, 394–400. 10.1111/jgh.12072, PMID: 23216461

[ref67] Vaziri N. D. (2014). Role of dyslipidemia in impairment of energy metabolism, oxidative stress, inflammation and cardiovascular disease in chronic kidney disease. Clin. Exp. Nephrol. 18, 265–268. 10.1007/s10157-013-0847-z, PMID: 23974528

[ref68] Wada T. Penninger J. M. (2004). Mitogen-activated protein kinases in apoptosis regulation. Oncogene 23, 2838–2849. 10.1038/sj.onc.1207556, 15077147

[ref69] Wahhabaghai H. Heidari R. Zeinoddini A. Soleyman-Jahi S. Golmanesh L. Rasoulian B.. (2015). Hyperoxia-induced preconditioning against renal ischemic injury is mediated by reactive oxygen species but not related to heat shock proteins 70 and 32. Surgery 157, 1014–1022. 10.1016/j.surg.2015.01.025, PMID: 25847506

[ref70] Wang H. E. Muntner P. Chertow G. M. Warnock D. G. (2012). Acute kidney injury and mortality in hospitalized patients. Am. J. Nephrol. 35, 349–355. 10.1159/000337487, PMID: 22473149 PMC3362180

[ref71] Wang Z. F. Tang L. L. Yan H. Wang Y. J. Tang X. C. (2006). Effects of huperzine a on memory deficits and neurotrophic factors production after transient cerebral ischemia and reperfusion in mice. Pharmacol. Biochem. Behav. 83, 603–611. 10.1016/j.pbb.2006.03.027, PMID: 16687166

[ref72] Wang L. Xue Y. Ma H. Shi H. Wang L. Cui X. (2017a). Prazosin protects myocardial cells against anoxia-reoxygenation injury via the extracellular signal-regulated kinase signaling pathway. Mol. Med. Rep. 17, 2145–2152. 10.3892/mmr.2017.8175 29207167 PMC5783458

[ref73] Wang A. Zhang H. Liang Z. Xu K. Qiu W. Tian Y.. (2016). U0126 attenuates ischemia/reperfusion-induced apoptosis and autophagy in myocardium through MEK/ERK/EGR-1 pathway. Eur. J. Pharmacol. 788, 280–285. 10.1016/j.ejphar.2016.06.038, PMID: 27343376

[ref74] Wang S. Zhao L. Li J. Luo Y. Han S. Zhang D. (2017b). cPKCc-mediated down-regulation of UCHL1 alleviates ischaemic neuronal injuries by decreasing autophagy via ERK-mTOR pathway. J. Cell. Mol. Med. 21, 3641–3657. 10.1111/jcmm.13275 28726275 PMC5706506

[ref75] Weyker P. D. Webb C. A. J. Kiamanesh D. Flynn B. C. (2013). Lung ischemia reperfusion injury: a bench-to-bedside review. Semin. Cardiothorac. Vasc. Anesth. 17, 28–43. 10.1177/1089253212458329, PMID: 23042205

[ref76] Widmann C. Gibson S. B. Jarpe M. B. Johnson G. L. (1999). Mitogen-activated protein kinase: conservation of a three-kinase module from yeast to human. Physiol. Rev. 79, 143–180. 10.1152/physrev.1999.79.1.143 9922370

[ref77] Wilson A. J. Gibson P. R. (1997). Epithelial migration in the colon: filling in the gaps. Clin. Sci. 93, 97–108. 10.1042/cs0930097, PMID: 9301423

[ref78] Wu S. Li M. Ko F. Wu G. Huang K. Chu S. (2013). Protective effect of hypercapnic acidosis in ischemia-reperfusion lung injury is attributable to upregulation of heme oxygenase-1. PLoS One 8:e74742. 10.1371/journal.pone.0074742, PMID: 24040332 PMC3769390

[ref79] Xu B. Gao X. Xu J. Lei S. Xia Z. Y. Xu Y.. (2011). Ischemic postconditioning attenuates lung reperfusion injury and reduces systemic proinflammatory cytokine release via heme oxygenase 1. J. Surg. Res. 166, e157–e164. 10.1016/j.jss.2010.11.902, PMID: 21227458

[ref80] Xu S. Niu P. Chen K. Xia Y. Yu Q. Liu N.. (2017). The liver protection of propylene glycol alginate sodium sulfate preconditioning against ischemia reperfusion injury: focusing mapk pathway activity. Sci. Rep. 7:15175. 10.1038/s41598-017-15521-3, PMID: 29123239 PMC5680172

[ref81] Yang M. Chen J. Zhao J. Meng M. (2014). Etanercept attenuates myocardial ischemia/reperfusion injury by decreasing inflammation and oxidative stress. PLoS One 9:e108024. 10.1371/journal.pone.0108024, PMID: 25260027 PMC4178063

[ref82] Yang Y. Hu S. J. Li L. Chen G. P. (2008). Cardioprotection by polysaccharide sulfate against ischemia/reperfusion injury in isolated rat hearts. Acta Pharmacol. Sin. 30, 54–60. 10.1038/aps.2008.12 19098935 PMC4006537

[ref83] Yndestad A. Sandanger O. Jong W. M. Aukrust P. Zuurbier C. J. (2016). Response to letter from Toldo et al. on “NLRP3 inflammasome activation during myocardial ischemia reperfusion is cardioprotective”. Biochem. Biophys. Res. Commun. 474, 328–329. 10.1016/j.bbrc.2016.04.096, PMID: 27109473

[ref84] Yu H. Shi L. Qi G. Zhao S. Gao Y. Li Y. (2016). Gypenoside protects cardiomyocytes against ischemia-reperfusion injury via the inhibition of mitogen-activated protein kinase mediated nuclear factor kappa b pathway in vitro and in vivo. Front. Pharmacol. 7:148. 10.3389/fphar.2016.00148, PMID: 27313532 PMC4887463

[ref85] Yu W. Xu M. Zhang T. Zhang Q. Zou C. (2019). Mst1 promotes cardiac ischemia–reperfusion injury by inhibiting the erk-creb pathway and repressing fundc1-mediated mitophagy. J. Physiol. Sci. 69, 1–15. 10.1007/s12576-018-0627-3 PMC10717665 29961191

[ref86] Zaleśna I. Hartman M. L. Czyz M. (2016). BRAF mutation in progression and therapy of melanoma, papillary thyroid carcinoma and colorectal adenocarcinoma. Postepy Hig. Med. Dosw. 70, 471–488. 10.5604/17322693.1201719, PMID: 27180965

[ref87] Zhang J. Xia J. Zhang Y. Xiao F. Li J. (2016). Hmgb1-tlr4 signaling participates in renal ischemia reperfusion injury and could be attenuated by dexamethasone-mediated inhibition of the erk/nf-κb pathway. Am. J. Transl. Res. 8, 4054–4067. PMID: . 27829992 PMC5095301

[ref88] Zhang Z. Yu J. (2018). NR4A1 promotes cerebral ischemia reperfusion injury by repressing Mfn2-mediated mitophagy and inactivating the MAPK-ERK-CREB signaling pathway. Neurochem. Res. 43, 1963–1977. 10.1007/s11064-018-2618-4, PMID: 30136162

[ref89] Zhou Q. L. Teng F. Zhang Y. S. Sun Q. Cao Y. X. Meng G. W. (2018). Fpr1 gene silencing suppresses cardiomyocyte apoptosis and ventricular remodeling in rats with ischemia/reperfusion injury through the inhibition of mapk signaling pathway. Exp. Cell Res. 370, 506–518. 10.1016/j.yexcr.2018.07.016, PMID: 30031130

[ref90] Zhu B. L. Xie C. L. Hu N. N. Zhu X. Liu C. (2018). Inhibiting of GRASP65 phosphorylation by DL-3-N-Butylphthalide protects against cerebral ischemia-reperfusion injury via ERK Signaling. Behav. Neurol. 2018, 1–7. 10.1155/2018/5701719, PMID: 30154935 PMC6093058

[ref91] Zuo Z. Wang Y. Huang Y. (2006). Isoflurane preconditioning protects human neuroblastoma sh-sy5y cells against in vitro simulated ischemia-reperfusion through the activation of extracellular signal-regulated kinases pathway. Eur. J. Pharmacol. 542, 84–91. 10.1016/j.ejphar.2006.05.027, PMID: 16806162

